# Editorial: From palm to plate, from awareness to action – toward sustainable palm oil supply chains and consumption

**DOI:** 10.3389/fnut.2025.1671825

**Published:** 2025-08-19

**Authors:** Sui Kiat Chang, Ágoston Temesi, Brad Ridoutt

**Affiliations:** ^1^Department of Allied Health Sciences, Faculty of Science, Universiti Tunku Abdul Rahman, Jalan Universiti, Bandar Barat, Kampar, Perak, Malaysia; ^2^Centre for Biomedical and Nutrition Research, Universiti Tunku Abdul Rahman, Jalan Universiti, Bandar Barat, Kampar, Perak, Malaysia; ^3^Department of Agricultural Business and Economics, Institute of Agricultural and Food Economics, Hungarian University of Agriculture and Life Sciences, Gödöllő, Hungary; ^4^Commonwealth Scientific and Industrial Research Organisation (CSIRO), Clayton, VIC, Australia; ^5^Department of Agricultural Economics, University of the Free State, Bloemfontein, South Africa

**Keywords:** palm oil, sustainability, consumption, production, environment

The UN Sustainable Development Goals offer a comprehensive summary of global problems of the modern world and their solutions, but the practical realization of these is a much more complicated issue (1). A striking example of this is the palm oil production system. On the one hand, the rather contradictory consequences of palm oil production on the environment are well-documented and known in academia. In fact, the increasing production and export are a strategic question for economic development and sovereignty for numerous developing countries, generating resources for the socio-economic catch-up to the developed countries (2). Palm oil has a few applications in giving foods desirable characteristics, such as improved shelf-life and thermal stability, and may offer certain nutritional health benefits. Moreover, in non-nutritive applications, palm oil has numerous uses in beauty products and cosmetics, cleaning agents, biofuels, and more (3).

This is why the palm oil production and consumption topic is very complex and can be analyzed only on the basis of a system approach, taking into consideration a wide array of factors influencing the actual behavior of different actors. Some palm-oil-producing countries have developed a system for the promotion of sustainable production practices (“Roundtable for Sustainable Palm Oil,” RSPO), but the level of knowledge and influence of this system on consumer behavior and purchasing decisions is unknown (4). Currently, there is a lack of such a comprehensive platform, where the various results of research on different aspects, from production through trade to consumption, of palm oil production could be presented. The goal of this Research Topic is to create a channel of communication for palm-oil-related research results, applying a holistic approach based on the “farm to fork” concept.

In this Research Topic, there are four papers covering the above-mentioned aspects. The first paper by Ostfeld and Reiner highlights the significant environmental impact of palm oil production, particularly in Indonesia and Malaysia, due to deforestation. Palm oil is a crucial component in many consumer products, driven by its widespread use as a replacement for hydrogenated oils. Palm oil production has been linked to significant deforestation and ecosystem loss in Southeast Asia. The authors emphasize the importance of sustainable palm oil production and the role of certification schemes, such as the RSPO, which aim to improve the environmental sustainability of palm oil production in mitigating environmental impacts. The recent EU regulation on deforestation-free products has implications for the palm oil industry and other forest products. This paper also highlights the potential of the RSPO as a model for other commodities subject to the new EU regulation. In summary, this paper emphasizes the urgent need for sustainable palm oil production practices and the crucial role of certification schemes in mitigating the environmental and social impacts of this globally important commodity (Ostfeld and Reiner).

The second paper by Sundaraja et al. reports that despite consumer intentions to purchase Sustainable Palm Oil (SPO), there is a significant gap between their intentions and actual purchasing behavior. While campaigns can increase consumer knowledge and motivation, they often fail to address practical barriers to purchasing SPO, such as limited availability and difficulty in identifying SPO products. Consumers may underestimate the challenges in finding and identifying SPO products in the market. Sundaraja et al. highlight the importance of the Capability-Opportunity Motivation model of Behavior (COM-B) as a framework that provides a valuable lens for understanding the factors influencing consumer behavior regarding SPO purchases. The article argues that while consumer behavior is important, it is insufficient to drive industry-wide change. This article emphasizes the need for increased corporate responsibility in utilizing SPO, improving product availability, and enhancing product labeling. The article suggests that national procurement policies for SPO can play a crucial role in driving long-term change. In essence, the article argues that while consumer awareness and intention are important, addressing the “opportunity” aspect of the COM-B model, through increased corporate responsibility and government policies, is critical for bridging the intention-behavior gap and promoting the widespread adoption of sustainable palm oil (Sundaraja et al.).

The third paper by Acobta et al. synthesizes a wide range of literature to provide a comprehensive overview that oil palm cultivation in Cameroon had significant negative impacts on various ecosystem services, particularly carbon sequestration, habitat quality, and genetic diversity. While oil palm cultivation provides food, it comes at the cost of significant losses in other crucial ecosystem services. Current policy responses primarily address environmental impacts, such as deforestation, while overlooking many significant social impacts. The paper by Acobta et al. addresses the lack of research on the social impacts of the palm oil trade in Africa, particularly in Cameroon. Hence, there is a need to address social impacts alongside environmental impacts in policy responses to the palm oil challenges. Findings of this study can inform the development of more effective and comprehensive policies for sustainable palm oil production and trade, both in Cameroon and other regions (Acobta et al.).

The fourth paper by Kovács et al. tested a mixture of fully hydrogenated (FH) rapeseed oil and sunflower oil as a potential substitute for palm oil. Authors reported that the 35% FH rapeseed oil blend showed rheological properties closest to palm mid-fraction (PMF), the control sample. This indicates that the 35% FH rapeseed blend could be a partial substitute in food applications where rheology is critical (e.g., spreads and baked goods). While some blends mimicked palm oil's behavior, the study concluded that full replacement is only viable to a limited extent from a rheological perspective. Further research is needed to optimize cost, scalability, and sensory properties for commercial use. These results addressed the urgent need for sustainable alternatives to palm oil due to its deforestation and ecological concerns as outlined by Ostfeld and Reiner. In short, the food industry cannot fully abandon palm oil yet, but this study identifies a promising partial alternative while underscoring the complex trade-offs between sustainability, functionality, and economics.

To summarize, the results of the above-mentioned studies and reviews represent some new relevant data on the palm-oil-related research results, applying a holistic approach based on the “farm to fork” approach. Despite all the existing literature and evidence related to this important topic, the papers published in this Research Topic clearly show that there are still many aspects to be understood in the studies related to palm oil and the environment. By reading this Research Topic, some topics, such as the environmental impact of palm oil production and cultivation, consumer intentions to purchase sustainable palm oil, and sustainable alternatives to palm oil, will be clearer to the scientific community and reinforce the important roles of palm oil. The scientific community is expecting more exciting results from palm-oil-related research in the coming years.
